# Surgical treatments for postamputation pain: study protocol for an international, double-blind, randomised controlled trial

**DOI:** 10.1186/s13063-023-07286-0

**Published:** 2023-05-02

**Authors:** Emily Pettersen, Paolo Sassu, Carina Reinholdt, Peter Dahm, Ola Rolfson, Anders Björkman, Marco Innocenti, Francesca Alice Pedrini, Juan Manuel Breyer, Aidan Roche, Andrew Hart, Lorraine Harrington, Adil Ladak, Hollie Power, Jacqueline Hebert, Max Ortiz-Catalan

**Affiliations:** 1 Center for Bionics and Pain Research, Mölndal, Sweden; 2 Department of Electrical Engineering, Chalmers University of Technology, Gothenburg, Sweden; 3 Center for Advanced Reconstruction of Extremities, Sahlgrenska University Hospital, Mölndal, Sweden; 4 Department of Orthoplastic, IRCCS Istituto Ortopedico Rizzoli, Bologna, Italy; 5 Department of Hand Surgery, Institute of Clinical Sciences, Sahlgrenska Academy, University of Gothenburg, Sahlgrenska University Hospital, Gothenburg, Sweden; 6 Department of Anaesthesia and Intensive Care, Institute of Clinical Sciences, Sahlgrenska Academy, University of Gothenburg, Sahlgrenska University Hospital, Gothenburg, Sweden; 7 Department of Orthopaedics, Institute of Clinical Sciences, Sahlgrenska Academy, University of Gothenburg, Gothenburg, Sweden; 8 Department of Orthoplastic, IRCCS Istituto Ortopedico Rizzoli, University of Bologna, Bologna, Italy; 9 Department of Orthopaedic, Hand Unit, Worker Hospital, Santiago, Chile; 10 College of Medicine and Veterinary Medicine, The Queen’s Medical Research Institute, The University of Edinburgh, Edinburgh, UK; 11 Canniesburn Plastic Surgery Unit, Glasgow Royal Infirmary, 84 Castle Street, Glasgow, G40SF UK; 12 College of Medicine, Veterinary & Life Sciences, The University of Glasgow, University Avenue, Glasgow, G12 8QQ UK; 13 Department of Anaesthesia, St John’s Hospital at Howden, NHS Lothian, Livingston, UK; 14 Division of Plastic Surgery, Department of Surgery, Faculty of Medicine and Dentistry, University of Alberta, Edmonton, AB Canada; 15 Department of Medicine, University of Alberta, Edmonton, AB Canada; 16 Bionics Institute, Melbourne, Australia

**Keywords:** Residual limb pain, Stump pain, Neuroma pain, Phantom limb pain, Targeted muscle reinnervation, Regenerative peripheral nerve interfaces, Randomised controlled trial

## Abstract

**Background:**

Painful conditions such as residual limb pain (RLP) and phantom limb pain (PLP) can manifest after amputation. The mechanisms underlying such postamputation pains are diverse and should be addressed accordingly. Different surgical treatment methods have shown potential for alleviating RLP due to neuroma formation — commonly known as neuroma pain — and to a lesser degree PLP. Two reconstructive surgical interventions, namely targeted muscle reinnervation (TMR) and regenerative peripheral nerve interface (RPNI), are gaining popularity in postamputation pain treatment with promising results. However, these two methods have not been directly compared in a randomised controlled trial (RCT). Here, we present a study protocol for an international, double-blind, RCT to assess the effectiveness of TMR, RPNI, and a non-reconstructive procedure called neuroma transposition (active control) in alleviating RLP, neuroma pain, and PLP.

**Methods:**

One hundred ten upper and lower limb amputees suffering from RLP will be recruited and assigned randomly to one of the surgical interventions (TMR, RPNI, or neuroma transposition) in an equal allocation ratio. Complete evaluations will be performed during a baseline period prior to the surgical intervention, and follow-ups will be conducted in short term (1, 3, 6, and 12 months post-surgery) and in long term (2 and 4 years post-surgery). After the 12-month follow-up, the study will be unblinded for the evaluator and the participants. If the participant is unsatisfied with the outcome of the treatment at that time, further treatment including one of the other procedures will be discussed in consultation with the clinical investigator at that site.

**Discussion:**

A double-blind RCT is necessary for the establishment of evidence-based procedures, hence the motivation for this work. In addition, studies on pain are challenging due to the subjectivity of the experience and the lack of objective evaluation methods. Here, we mitigate this problem by including different pain evaluation methods known to have clinical relevance. We plan to analyse the primary variable, mean change in NRS (0–10) between baseline and the 12-month follow-up, using the intention-to-treat (ITT) approach to minimise bias and keep the advantage of randomisation. The secondary outcomes will be analysed on both ITT and per-protocol (PP). An adherence protocol (PP population) analysis will be used for estimating a more realistic effect of treatment.

**Trial registration:**

ClinicalTrials.gov NCT05009394.

**Supplementary Information:**

The online version contains supplementary material available at 10.1186/s13063-023-07286-0.

## Background

**Fig. 1:**
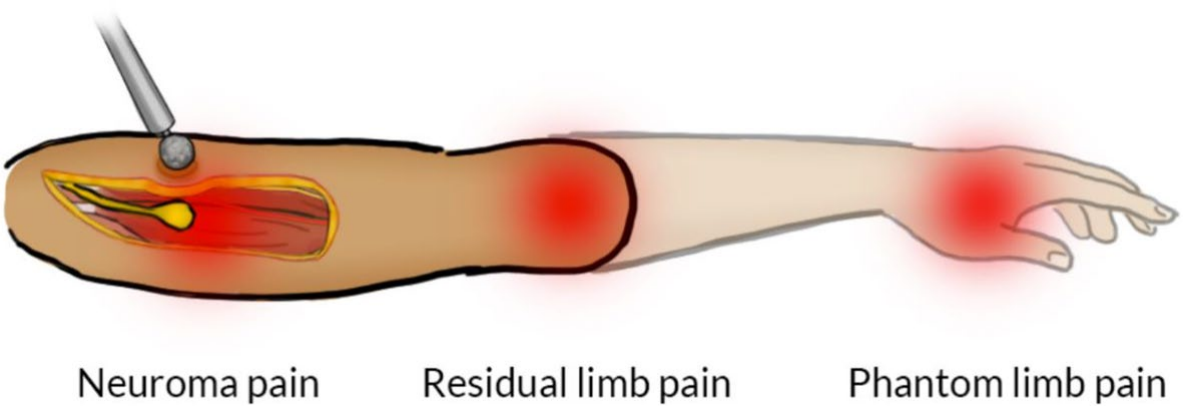
Illustration of the three different pain types: neuroma pain, residual limb pain, and phantom limb pain. Note that neuroma and residual limb pain can be felt anywhere in the residual limb

In addition to the loss of function, the amputation of extremities often results in neuropathic pain [1, 2]. The International Association for the Study of Pain (IASP) makes a distinction between pain localised in the residual limb or stump (residual limb pain (RLP)) and pain perceived in the missing or phantom limb (phantom limb pain (PLP)) [3]. The underlying causes of such postamputation pains are diverse and must be addressed accordingly [4]. For instance, an amputation severs the nerves that generate sprouting of axons resulting in a neuroma. A neuroma is a disorganised bulge at the distal end of the nerve comprising axons, Schwann cells, and endo- and peri-neural cells, all within a dense myofibroblast stroma [5]. The IASP considers neuromas as a source of RLP along with muscle, bone, and other sensory abnormalities such as hypoesthesia, allodynia, and hyperalgesia [6]. Mechanical stimulation of neuromas is normally painful and can also elicit phantom limb pain (PLP) according to IASP as their definition is based on the location of perceived pain. PLP can be elicited by stimulation of nerve fibres that previously innervated nociceptors in the missing limb and are now trapped within the neuroma (“nociceptive PLP”), as well as due to maladaptive plastic changes in the nervous system (“neuropathic PLP”) [4]. For example, the successful treatment of neuromas does not always resolve PLP [7]. Similarly, whereas neuromas can be a source of RLP, they are not the only one [8]. For these reasons, here, we consider neuroma pain—a focal aggravation suggestive of a neuroma—as a subtype of RLP and PLP, which can be identified distinctively by mechanical stimulation. In contrast, RLP and PLP will be identified as pains perceived in the residual and phantom limbs, respectively, and without mechanical stimulation (Fig. 1).

Historically, non-surgical treatments of postamputation pain were considered more effective than surgical treatment options [9]. However, this has changed in the last decade with the development of surgical approaches that prevent the formation of neuromas by allowing severed nerves to grow into new target muscles [10]. Two reconstruction techniques have gained popularity for the treatment of neuroma pain, targeted muscle reinnervation (TMR) and regenerative peripheral nerve interface (RPNI). Both techniques are based on similar biological principles—reinnervation of denervated muscle—but there are important differences [11]. In TMR, the residual peripheral nerve is transferred to the stump of a nerve innervating a native muscle with a dispensable biomechanical function [12]. The RPNI procedure involves longitudinally dissecting the severed nerve into its main fascicles, which are then implanted into free muscle grafts [13, 14]. TMR was developed with the initial aim to increase the prosthetic function of a myoelectric artificial limb [15, 16]. However, in an early clinical series, it was observed that patients undergoing TMR surgery experienced a notable pain reduction and did not report new painful neuromas [17]. Pain reduction was later reported in a randomised controlled trial (RCT) in which a non-reconstructive active control was employed [12]. The non-reconstructive procedure (neuroma transposition) entailed dissecting the neuroma and burying the distal end of the nerve into a muscle without denervating it. Similarly, in a retrospective clinical case series, patients reported decreased neuroma pain and PLP after undergoing RPNI surgery [13]. Despite the ongoing widespread use of these two surgical techniques, clinical trials comparing the two procedures are missing.

### Study objective

Here, we present the clinical study protocol for a double-blind RCT in which lower and upper extremity amputees are treated for postamputation pain with three surgical approaches. The main objective is to assess the effectiveness of nerve reconstruction techniques in treating postamputation pain. In addition to treatment with TMR or RPNI, we use a previously employed active control whereby severed nerves are surgically inserted into a remanent muscle that retains its original innervation (unlike TMR where the muscle is denervated from its native nerve).

### Trial design

**Fig. 2:**
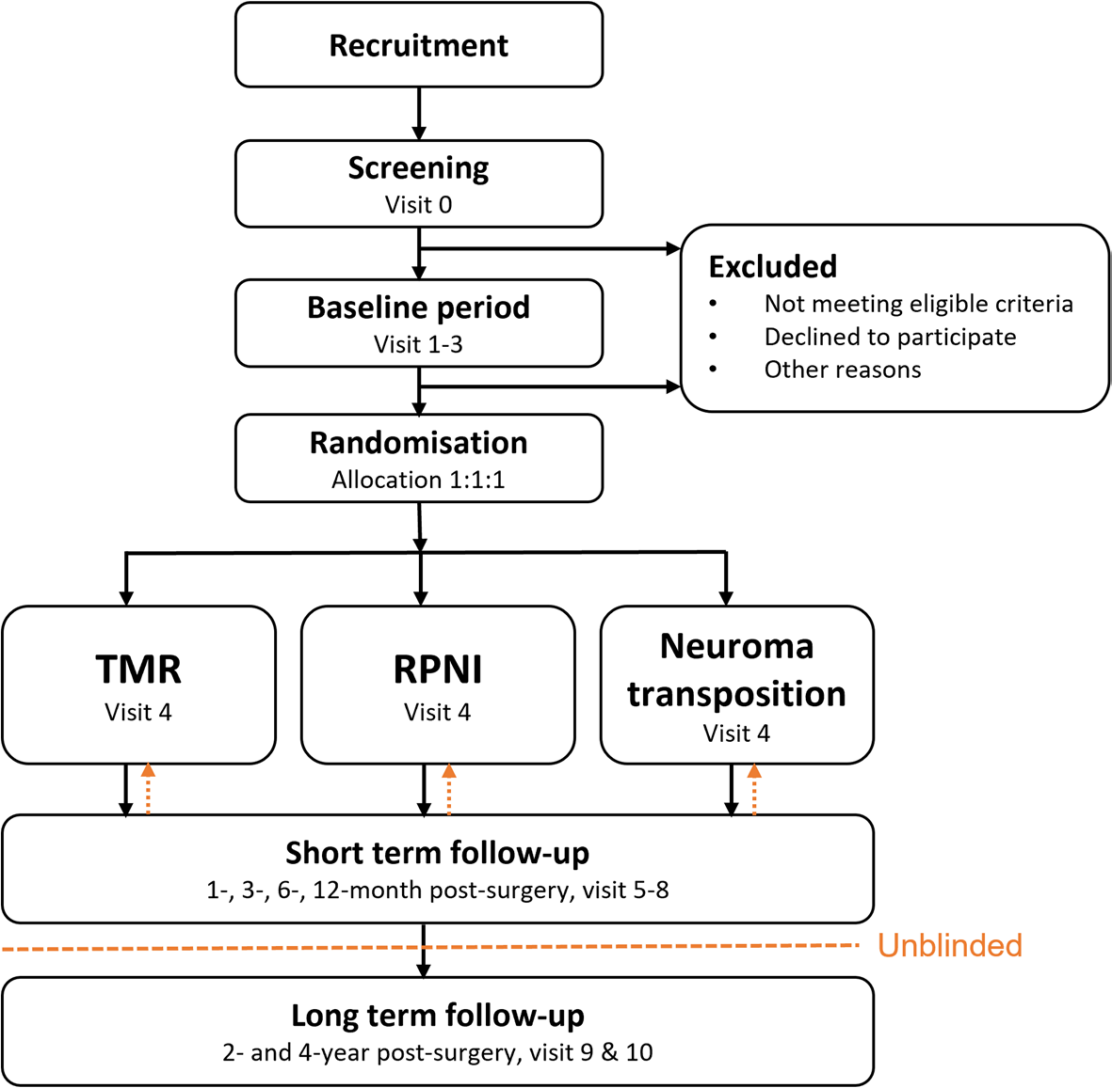
Study flowchart of the double-blind, randomised controlled trial. One hundred ten lower and upper amputees are recruited and randomly assigned to one of the surgical treatments for postamputation pain in an equal allocation ratio. The follow-up is short term (1 to 12 months post-surgery) as well as long term (2 and 4 years post-surgery). The study will be unblinded 12 months post-surgery, where treatment outcomes will be discussed in consultation with the clinical investigator at the site

This is a multi-centre, double-blind, superiority RCT which takes place at 9 hospitals in 7 countries:the Sahlgrenska University Hospital in Gothenburg, Sweden; the Rizzoli Orthopaedic Institute in Bologna, Italy; the University of Alberta Hospital in Edmonton, Canada; Worker Hospital in Santiago, Chile; the NHS Lothian, NHS Clyde & Greater Glasgow, and NHS Grampian, UK; Dandenong Hospital, Monash Health in Melbourne, Australia; the Northwestern Memorial Hospital in Chicago, USA; the University of Michigan Health System in Ann Arbor, Michigan, USA; and within the Massachusetts General Hospital in Boston, USA. One hundred ten participants will be recruited and randomly assigned to one of three surgical treatments (TMR, RPNI, or neuroma transposition) in an equal allocation ratio (*n* = 37 per group). Each participant will be followed up short term (1, 3, 6, and 12 months post-surgery) and long term (2 and 4 years post-surgery). After the 12-month follow-up, the study will be unblinded for the evaluator and the participants. If the participants are unsatisfied with the outcome of the treatment, they may request one of the other treatments. In such a case, a medical evaluation and further treatment options will be discussed in consultation with the clinical investigator at the site. See the study flowchart in Fig. 2.

## Methods/design

### Participants

#### Recruitment and inclusion of participants

Potential participants will be identified and recruited by healthcare professionals or among people who have had earlier contact with the research groups. Clinical trial brochures will be distributed to prosthetic clinics and orthopaedic and hand surgery departments. The trial will be advertised on social media, in local newspapers, and at national conferences in Sweden, Italy, Canada, Chile, Australia, UK, and USA. Persons interested in participating will be provided with written information about the study, and they will be directed to a pre-screening form either in paper or digital format (Additional file 1). After submission of the pre-screening form, the potential participant will be contacted by phone or via email. If the potential participant is interested in participating in the study, the participant will be provided with an informed consent form (ICF) (Additional file 2) and invited to a screening visit. At the screening visit, the clinical investigator on site will perform a physical examination, and the participants answer any questions. Answers to the pre-screening form will be used in combination with the screening visit to determine eligibility for the study. The informed consent form will be signed by the participant and the site clinical investigator before collecting any study-specific data. The following are the study eligibility criteria.

The following are the inclusion criteria:

- The participant must have a major limb amputation.
- The participant is ≥ 18 years old at the time of consent.
- The participant must be in generally good health to undergo a surgical intervention, as per the clinical investigator’s opinion.
- Time since the last amputation must be over a year at the time of consent.
- The participant must have an average residual limb pain score equal or greater than 4 on the Numerical Rating Scale (NRS, 0–10) after the baseline period.
- If the participant has been prescribed pharmacological treatments for pain, there must be no variations in dosage (steady consumption) for at least 1 month before the screening visit.
- If the participant has been prescribed non-pharmacological treatments for pain, such as spinal cord stimulation, transcutaneous electrical nerve stimulation, and mirror therapy, the treatment must have ended at least 1 month before the screening visit.
- The participant must have a stable prosthetic fitting for at least a month before the screening visit.
- The participant has a sufficient understanding of the language in which the assessments will be conducted, as per the clinical investigator’s opinion.

The following are the exclusion criteria:

- Neurological or other conditions that affect nerve regeneration for the nerve to be treated.
- Active infection in the residual limb.
- Prior RPNI or TMR surgery of the nerve to be treated (with painful neuroma) to address postamputation pain.
- Mental disorders (e.g., schizophrenia, paranoia, psychosis, etc.), reluctance, or language difficulties that result in difficulty understanding the meaning of study participation.
- Ongoing participation in a clinical study that the clinical investigator deems detrimental to participation in this study.

During this clinical trial, participants will not be forbidden to receive other pain treatments if deemed necessary by their physician. All pain treatments received during the clinical trial will be recorded.

#### Withdrawal or termination of individual participants

Participants can discontinue their participation in the study at any time, without any consequences on their clinical treatment at their respective hospitals or health care systems. The clinical investigator at the site can at any time terminate participation in the study for an individual if the participant’s safety can no longer be maintained due to any harmful clinical event, clinical anomalies, if other medical conditions or situations occur, or if the participant no longer fulfils the study criteria due to recently developed or not previously recognised disorders. Adverse events (AE) will be presented together with the results of the study.

We will employ the intention-to-treat (ITT) methodology in the analysis of the primary outcome. In case of non-adherence to the protocol, such as crossovers between surgical interventions after randomisation, the data will be analysed by grouping participants to their originally allocated intervention. Furthermore, the last collected data point will be used in the primary analysis in the case that data is missing due withdrawal, termination, or missed follow-ups. A sensitivity analysis of the primary analysis will be performed. A complementary analysis of the primary outcome will also be performed on the per-protocol (PP) population. The secondary outcomes will be analyses on both ITT and PP populations. Based on previous studies, drop-outs are expected to be around 10%.

### Interventions

The surgical interventions take approximately 1 to 3 h each, and the participant will stay minimum 12 hours after surgery, unless the clinical investigator or surgeon in charge at the site decides that the participant needs to stay longer for observation. The anaesthesia will be managed by a intravenous approach and locoregional anesthesia or general anesthesia will be used without muscle relaxants in TMR. General pre-surgical rules will apply, i.e. abstaining from alcohol for 4 weeks before surgery and fasting from midnight the night before surgery. There will not be any post-surgery restrictions such as immobilisation of the residual limb and regular dressing will be used. Participants will receive post-surgical continuous pain medication through a catheter minimum 12 hours after surgery and pain rehabilitation instructions on how to perform mirror therapy at home (Additional file 3).

#### Targeted muscle reinnervation (TMR)

**Fig. 3:**
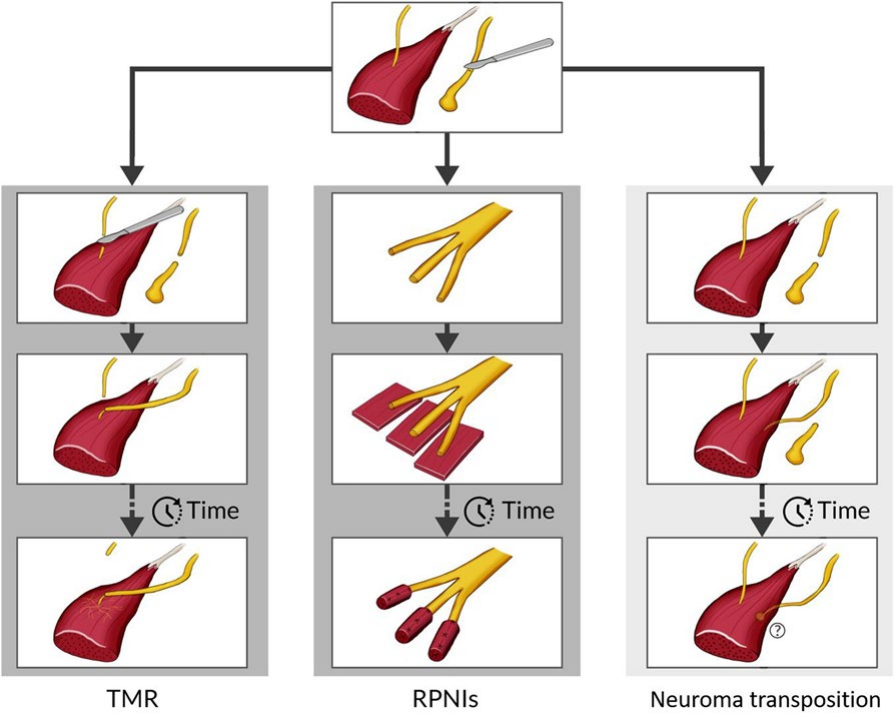
Schematic illustration of the three surgical approaches included in the trial: targeted muscle reinnervation (TMR), regenerative peripheral nerve interface (RPNI), and standard treatment (neuroma transposition)

The TMR procedure for pain management is described in detail in the published literature [7, 17, 18]. Briefly, TMR is a surgical technique in which the residual peripheral nerve is transferred to an available muscle in the residual limb that has been denervated from its native nerve (Fig. 3, left). The surgical procedure comprises three steps: firstly preparation of the donor nerve, secondly identification of a motor branch to the targeted muscle, and finally, nerve coaptation [18]. To prepare the donor nerve, the surgeon will identify the nerve with a painful neuroma and resect the neuroma up to healthy fascicles. Next, the surgeon will identify a motor branch to a nearby target muscle and will confirm muscle contraction using a hand-held nerve stimulator. If several motor branches are identified, the motor branch with the largest muscle contraction will be used. The motor branch to the target muscle will be transected as close as possible (aiming for less than 1 cm) to its entry point without tension, and thereby temporarily denervating the target muscle. In the final step, the previously nerve stump from which the neuroma was resected will be transferred and coapted to the newly severed motor branch that innervates the target muscle and secured by 2-3 non-resorbable monofilament sutures [18].

#### Regenerative peripheral nerve interface (RPNI)

A detailed description of the RPNI surgery has previously been described in the literature [11, 13, 14, 19]. Briefly, the RPNI procedure involves splitting the residual peripheral nerve into several nerve fascicles which are implanted into skeletal muscle grafts [19] (Fig. 3, middle). The surgeon will first identify the nerve with a painful neuroma and resect the neuroma up to healthy fascicles [14]. Then, a longitudinal intraneural dissection for about 2 cm will be performed exposing its fascicles. Next, autologous muscle grafts will be harvested from a healthy donor site, and the dissected nerve stumps will be placed in the middle part of each muscle graft, parallel to the muscle fibres. The nerve stump will be secured to the muscle graft by proximally and distally non-resorbable monofilament sutures and thereafter the muscle graft will be wrapped around the nerve stump and anchored by non-resorbable monofilament sutures in the base and the walls of the folded graft, thus creating an RPNI. This will be repeated for each fascicle obtained from splitting the transected nerve [14]. Lastly, the RPNIs will be placed in a protected area where each RPNI will lie comfortably and out of weight-bearing surfaces of the limb. 

#### Standard neuroma treatment, neuroma excision, and muscle burying

The standard neuroma treatment entails the excision of the terminal neuroma and then implanting the nerve into an adjacent muscle (Fig. 3, right). Firstly, the surgeon will identify the nerve with a painful neuroma and thereafter resect the neuroma up to healthy fascicles. Next, the surgeon will identify a nearby muscle which is not involved in joint motion and has limited output opportunities for the nerve. The nerve will then be channelled at least 1 cm inside the muscle without applying any tension to it and secured by 1-2 non-resorbable monofilament sutures [12]. The identified nerve with the painful neuroma will not be treated with any additional therapy than the resection ﻿(e.g., diathermy, pharmacotherapy, crushing, etc.).

### Outcomes

#### Primary outcome measure

The primary outcome measure in this study is the mean difference of change in residual limb pain intensity measured by the difference in NRS score (0–10) between baseline (visits 0 to 3) and at the 1-year follow-up post-surgery visit.

#### Secondary outcome measures

The secondary outcome measures include the mean difference of change for neuroma pain and phantom limb pain intensity measured in NRS score (0–10) between baseline (visits 0 to 3) and at the 1-year follow-up. Secondary outcomes also consider aspects such as pain frequency, duration, interference, and quality (Table 1) and changes that can cause variations in any of the pain types (Table 2).

**Table 1:** Questionnaires included in the study divided between residual limb pain, neuroma pain, phantom limb pain, and general pain

Questionnaire	Residual limp pain	Neuroma pain	Phantom limb pain	General pain
Pain survey				
NRS	X	X	X	
Pain frequency	X	X	X	
Pain rating index (PRI)	X	X	X	
Pain localisation			X	
Weighted pain distribution			X	
Intrusion of pain				X
Pain Disability Index (PDI)				X
EuroQoL (EQ-5D-5L)				X
Pain Self-Efficacy Questionnaire (PSEQ-2)				X
Pain Catastrophising Scale-6 (PCS-6)				X
Patients’ global impression of change (PGIC)				X
Expectations for Complementary and Alternative Medicine Treatments (EXPECT-SF)				X

**Table 2:** Variables that can potentially modulate postamputation pain

Pain survey
Phantom sensation (NRS)
Phantom motor ability
Phantom telescoping
Change in prosthetic situation
Change in medication

#### Distinguishing residual limb pain, neuroma pain, and phantom limb pain

Changes in residual limb pain, neuroma pain, and phantom limb pain will be investigated in this study. These three types of pain will be distinguished by taking advantage of fundamental differences in their perceived location and quality. Residual limb pain and phantom limb pain can be distinguished by the location of the pain, i.e. perceived in the residual limb or in the missing limb, respectively [3] (Fig. 1). For neuroma pain, physical stimulation in the form of digital pressure (e.g. Tinel’s sign test) will be used to elicit pain where the neuroma is suspected. No physical stimulation will be used to elicit residual limb pain or PLP; rather, participants will be asked to close their eyes and localise the pain in order to distinguish between the two types. In cases where gentle skin indentation results in RLP, the area can be locally anaesthetised to reduce it and uncover neuroma pain during the physical stimulation. Neuroma pain can also be distinguished by the distally referred painful sensations it elicits in the phantom limb during physical stimulation. At least one of the following methods should be applied to document the presence of neuroma pain: compatible symptomatology, Tinel's sign test, imaging (ultrasound or MRI), or nerve block.

##### Pain survey

The pain survey has 22 items based on the SF-MPQ [20] and study-specific questions. It is divided into five sections: residual limb pain, neuroma pain, phantom limb pain, general pain interference, and other questions. The survey evaluates the intensity, quality, and frequency of residual limb pain, neuroma pain, and phantom limb pain as follows:

- Numerical Rating Scale to evaluate the present intensity of pain (0–10 where 0 = no pain and 10 = worst imaginable pain).
- Present Pain Intensity Scale (range 0–5 calibrated as 0 = no pain, 1 = mild, 2 = discomforting, 3 = distressing, 4 = horrible, 5 = excruciating) [20].
- Pain quality is measured by SF-MPQ Pain Rating Index (PRI) by summing the 15 descriptions (range 0–3 calibrated as 0 = no pain, 1 = mild, 2 = moderate, 3 = severe) [20].
- Study-specific scale for evaluation of pain frequency (“constantly”, “few times per day”, “once a day”, “few times per week”, “once a week”, “few times per month”, “once a month”, “never”).

In addition, the phantom limb pain section monitors weighted pain distribution, phantom pain location, phantom telescoping, phantom sensation, and phantom motor ability [21].

The general pain interference questions track pain intrusion in daily activities, work, and sleep, using a numerical rating scale (0–10 where 0 = no interference and 10 = full interference) for each activity. Adjustment in prosthetic hardware and pain medication is also monitored in this section.

##### Pain disability index (PDI)

The PDI is a seven-item questionnaire which measures the extent to which chronic pain interferes with participation in activities of daily life [22]. Each question includes a life activity and the degree to which a person’s ability to perform the task is hindered (disability) by their pain and is rated from 0 to 10 where 0 = no disability and 10 = worst disability. The PDI score is calculated by summing the numerical ratings.

##### EuroQoL-5D-5L (EQ-5D-5L)

The questionnaire EQ-5D-5L (EuroQol Group) is a standardised survey used to evaluate health-related quality of life, in terms of health status and health evaluation [23]. The health status is measured and evaluated in five categories: mobility, self-care, usual activities, pain/discomfort, and anxiety/depression. Each category is rated by a five-item scale (no problems, slight problems, moderate problems, severe problems, and extreme problems). The health evaluation part includes the EQ visual analogue scale (VAS) (0–100 where 0 = worst imaginable health state, 100 = best imaginable health state), and the participant is asked to rate their health that particular day.

##### Pain self-efficacy questionnaire (PSEQ-2)

PSEQ-2 is a two-item questionnaire that rates pain self-efficacy, which is defined as the confidence held by people living with chronic pain that they can participate in certain activities and enjoy life, despite the pain they experience [24, 25]. The self-efficacy in each question is rated on a numerical rating scale (0–6 where 0 = not at all confidence and 6 = completely confidence).

##### Pain catastrophising scale (PCS-6)

PCS-6 is a six-item questionnaire designed to monitor catastrophising thinking, in a rating scale from 0 to 4 [26, 27]. Pain catastrophising implies the negative cognitive-affective reply to pain and is correlated with pain severity, disability, and depressive symptoms as well as with inadequate adjustment to chronic pain [28, 29].

##### Patients’ global impression of change (PGIC)

PGIC is a single-item questionnaire used for the identification of a significant clinical change by rating the participant’s belief about the treatment’s effectiveness on a 7-point rating scale (1–7, where 1 = no change and 7 = a great deal better).

##### Additional measurements

In addition to the aforementioned questionnaires, the participants will be asked to provide background information about themselves, such as age, gender, type and dose of medication, date of amputation, amputation cause, type and usage of prosthesis, and previous treatments. Furthermore, an additional survey will be conducted regarding the participants’ expectancy of benefit by using the expectations for complementary and alternative medicine treatments (EXPECT-SF) [30]. Moreover, the participants will be asked to participate in brief, semi-structured interviews in order to understand how the participants have experienced the treatment and how it has affected their quality of life. See Table 3 for a summary of all study-specific assessments at each visit.

**Table 3:** Summary of study-specific assessments at each study-specific session

Session	Assessments
**Pre-screening**	• Patient information • Study consent • Background information
**Screening, visit 0**	• Pain survey • PDI • EQ5D-5L • PSEQ-2 • PCS-6 EXPECT-SF
**Baseline, visits 1–3**	NRS (0–10); RLP, neuroma pain, PLP
**Randomisation, allocation 1:1:1**	
**Surgery, visit 4**	• Surgery
**Follow-up, visits 5–10**	• Pain survey • PDI • EQ5D-5L • PSEQ-2 • PCS-6 • PGIC • Interview (visit 8)
*Short-term*	
1 month	
3 months	
6 months	
12 months	
Unblinded	
*Long-term*	
2 years	
4 years	

*PDI* Pain disability index, *EQ5D-5L* EuroQol-5D-5L, *PSEQ-2* Two-item Pain Self-Efficacy Questionnaire*, PCS-6* Six-item pain catastrophising scale, *EXPECT-SF* Expectations for complementary and alternative medicine treatments short form, *NRS* Numerical rating scale, *RLP* Residual limb pain, *PLP* Phantom limb pain, *PGIC* Patients’ global impression of change

### Assignment of intervention

#### Sample size

Sample size calculation is based on previous studies investigating the surgical treatments for neuroma pain [12, 13] and based on the limited number of available participants. To receive a minimum power of 80% from a Fisher’s non-parametric permutation test assuming a standard deviation of TMR and RPNI to be 2.2 and 3.3 for control [12] with a mean difference of 2.0 and at 5% significant level between the randomised groups in an allocation ratio of 2:1 (TMR and RPNI vs control), it is estimated that at least 84 (*n* = 28 per group) participants are required. With the same assumptions, but instead looking at one reconstruction technique compared to control in a ratio of 1:1 (TMR vs control, RPNI vs control), 99 participants (*n* = 33 per group) are needed to reach minimum power of 80% with a Fisher’s non-parametric permutation test with a mean difference of 2 NRS and at 5% significant level. The drop-out rate is expected to be around 10%; therefore, a total of 110 participants will be aimed to be randomised.

## Randomisation

Participants will be assigned to either of the three treatments according to the optimal allocation scheme of minimisation that aims at reducing the imbalance between the number of participants allocated to each treatment group. The randomisation proportion aims to be 1:1:1, with the same number of major limb amputations assigned to each group. The allocation aims to minimise the imbalance of the following factors:

- Level of amputation (upper and lower)
- Baseline residual limb pain based on the NRS (medium 4–6 and high 7–10)
- Investigation site (Sweden, Italy, Canada, Chile, Australia, UK, and USA)

The clinical randomisation of the participants will be performed using the randomisation module in REDCap [31], a tool for electronic capturing of research data. When a new recruitment is made at a site, and the participant meets the study criteria and the minimising factors of the participant are embedded in the software. The software will run a randomisation and allocate the participant to one of the treatment groups and a pseudonym code will be automatically assigned to the study participant.

### Blinding

The study is designed so that the participant and the evaluator are blinded to the surgical treatment the participant has received. The participants will undergo the same study-specific assessments despite their assigned surgical treatment. The only difference between the groups will be the surgical treatment, although the participants in the RPNI group will have an additional scar on the muscle donor site (inconceivable to the participant) compared to the TMR and neuroma transposition group. The participants will not have any expectation of surgical outcome superiority since the trial will be communicated as a comparison between three surgical treatments formerly described in the literature. The evaluator and the participant will be unaware of which surgical treatment the participant has received up to 12 months post-surgery. Thereafter, the study will be unblinded for the participants and the evaluator. The clinical investigator in each site will be aware of the treatment allocation, and they are excluded from the analyses. The raw data following the outcome assessments from each surgical treatment will have the same structure, which make it unfeasible to differentiate the group assignments without having the document that links the specific treatments to participants’ code numbers.

## Data collection, management, and analysis

### Data collection and management

The clinical investigators and the principal investigator will share the overall responsibility for the progress of the RCT. The trial coordinator will be in charge of ensuring that the RCT is conducted, recorded, and reported according to the clinical protocols, Good Clinical Practise (GCP), and regulatory requirements. The study will be monitored twice a year by a project independent monitor educated in GCP.

The evaluator at each site will oversee the case report forms (CRFs) and documents the outcomes during the RCT. The data in the CRFs will be identified by participant-specific code numbers. Data documentation will also be applicable for participants who have given written consent for participation, then underwent the baseline assessments but decided to leave the study. In the CRF, all items must be filled in, none must be empty. However, if data is missing or is unfeasible to collect, this should be documented as “not available” (NA), and an explanation must be recorded in the CRF.

The clinical investigator at each site will be in charge of recording any occurrences of adverse events (AE), in discussion with the participant at each visit, and documentation of the AE in the participant’s CRF. Every AE needs to be explained by duration (start and stop dates and times), severity, outcome, treatment, and relation to the surgical treatment (related or unrelated). The clinical investigator must report any incidence of an AE to the principal investigator and the trial coordinator in a timely manner (no later than three working days after the incident).

The data collected in this study will include images, sound recordings, questionnaires and interview answers, and medical data. Images and sound recordings will only be shared with the written consent of the participant. Participants will be able to choose whether images can be shown undoctored or with their faces blurred or cropped out. All data will be pseudonymised with a code consisting of two letters and three digits and all collected data will be assigned the coded identity.

All data gathered within this RCT will be stored digitally in accordance with GDPR requirements, on a password-protected server with restricted access using two-factor authorisation. Only the researchers who are directly involved in the study will have access to the data. The principal investigator will ensure that all relevant research personnel have access to the data. The document which couples the surgical technique that the study participant received to their unique code will be password-protected and saved separately. All information will be confidential and stored in accordance with GDPR and with the study participant’s written informed consent.

### Statistical analyses

The statistical analyses will be performed pairwise comparing the techniques in the following scheme:

1. Reconstruction techniques compared to control: TMR and RPNI vs control (2:1)
2. Individual comparison of each reconstruction technique compared to the control: TMR vs control (1:1) and RPNI vs control (1:1)
3. Comparison between the reconstructive techniques: TMR vs RPNI (1:1)

Statistical analysis for the primary outcome, the mean difference of change in NRS score (0–10) of residual limb pain between baseline and 1-year follow-up between the two reconstructive surgical methods and control (2:1), will be performed with an ANCOVA for comparison of independent means on ITT population at 5% significance level, adjusted for the stratification (minimisation) variables NRS pain at baseline, level of amputation, and site. Imputation will be performed with the last observation carried forward (LOCF). A sensitivity analysis will be performed using a two-sided non-parametric permutation test for comparison of independent means on the ITT population. A complementary analysis will be performed for the primary variable of the PP population. The main results will be the mean difference with 95% CI, effect size, and *p*-value.

Secondary analyses will be performed unadjusted, between the two groups on both ITT and PP-population with Fisher’s non-parametric permutation tests for continuous variables, with the Mantel–Haenszel chi-square test for ordered categorical variables, and with Fisher’s exact test for dichotomous variables and performed adjusted with ANCOVA for continuous variables and with multivariable logistic regression for dichotomous variables.

Exploratory analyses will be performed to investigate the differences in groups such as lower vs upper extremity, right vs left extremity, female vs male, reason for amputation, baseline NRS (medium 4–6, high 7–10), time since amputation, and age. These exploratory analyses will be done within each group and then pooled together. All significance tests will be two-sided and conducted at the 5% significance level. When half of the study population has passed the 1-year follow-up, an independent committee including a medical expert and a statistician will examine the data with regard to safety.

## Discussion

Investigation in pain-related studies is challenging due to an absence of objective evaluation methods. Pain is a subjective experience that is difficult to quantify independently among participants. In our study design, we have tried to mitigate this problem by using different protocols and questionnaires for pain tracking that have previously been used in clinical settings.

One of the main challenges in this RCT is to recruit a sufficient number of potential candidates meeting our inclusion criteria within an acceptable time. For example, it took over 3 years for Dumanian et al. (2019) to gather 28 participants who met their study criteria [12], and this study was performed in the USA (with a larger amputee population compared to Sweden, Italy, Canada, Chile, and the UK). According to Sweden’s amputation and prothesis registry for lower extremities (SwedeAmp) yearly report in 2019, the mean age of amputation for women is 78 and for men is 72 years old. Furthermore, 61% of all amputated women and 58% of all amputated men are in the range of 70–89 years old, and 84% of all Swedish amputees have either diabetes and/or vascular disease. We expect this to add a significant challenge in the volume of participants required. Our recruitment approach therefore includes close contact with prosthetic centres, orthopaedics, and hand surgeons in the participating hospitals.

The surgical treatment approach requires that the participants undergo surgery; thus, general surgical risk applies, including but not limited to those associated with anaesthesia, the possibility of superficial or deep bacterial infections, and delayed wound healing. Even though the surgical approaches have shown potential for postamputation pain reduction [12, 13], there is a risk that the treatment results in no relief or a worse pain situation for the participant. This may lead to a need for increased pain suppression medication.

This RCT is designed as a double-blind study, where both the participants and the evaluator are unaware of which surgical treatment has been prescribed. However, since RPNI involves a muscle graft from a donor site, the RPNI participants will have an additional scar compared to TMR and the neuroma transposition group. We discounted the option to add a sham scar to participants from the other groups on ethical grounds and instead designed the RCT such that participants will not be told detailed methodological differences between the surgeries, and we do not expect them to know which surgical treatment they received. The participants can find out which treatment they received by searching in the literature, but even in that unlikely case, they still would not know which one of the surgeries is the reconstructive one or the active control.

## Trial status

This clinical study was registered at ClinicalTrials.gov on 17 August 2021 with registration number NCT05009394. This is protocol version 4 (2022–12-21). Recruitment of participants is expected to take place in early 2023 and is expected to be completed in 2025.

### Supplementary Information

**Additional file 1.** Pre-screening questionnaire, pdf. The questionnaire contains general questions regarding amputation situation of the potential research participant including questions about amputation, prosthetic situation, pain, and general health.

**Additional file 2.** Information and consent form, pdf. Written information about the study that potential research participants interested in participation will receive and consent form that the research participant and clinical investigator sign.

**Additional file 3.** Mirror therapy protocol, pdf. Instructions on how to perform mirror therapy at home by Alberta University Hospital.

## Data Availability

The clinical investigators and the principal investigator have full access to all study-specific data, except for the document including the link between surgical treatment and participant’s code number. That document will only be accessible after the 1-year follow-up analyses. They have the responsibility for correct data storing and for the accuracy of the data analysis.

## Abbreviations

AE: Adverse event
CRF: Case report form
GCP: Good clinical practice
ICF: Informed consent form
LOCF: Last observation carried forward
ITT: Intension-to-treat
NRS: Numerical Rating Scale
PLP: Phantom limb pain
PP: Per-protocol
RCT: Randomised controlled trial
RPNI: Regenerative peripheral nerve interface
TMR: Targeted muscle reintervention

## References

[1] Hsu E, Cohen SP (2013). Postamputation pain: epidemiology, mechanisms, and treatment. J Pain Res Dove Press.

[2] Limakatso K, Bedwell GJ, Madden VJ, Parker R. The prevalence and risk factors for phantom limb pain in people with amputations: A systematic review and meta-analysis. PLoS ONE. 2020;15(10):e0240431. 10.1371/journal.pone.0240431. 10.1371/journal.pone.0240431 PMC7556495 33052924

[3] Schug SA, Lavand P, Barke A, Korwisi B, Rief W (2019). The IASP classification of chronic pain for ICD-11: chronic postsurgical or posttraumatic. Pain..

[4] Ortiz-Catalan M (2018). The stochastic entanglement and phantom motor execution hypotheses: a theoretical framework for the origin and treatment of phantom limb pain. Front Neurol..

[5] Lee M, Guyuron B. Postoperative neuromas. Nerves Nerve Inj. Elsevier Ltd.; 2015.

[6] International Association for the Study of Pain. IASP global year against neuropathic pain 2014–2015, postamputation pain. 2014 [cited 2022 Nov 28]. p. 2. Available from: https://www.aped-dor.org/images/FactSheets/DorNeuropatica/en/Postamputation_Pain.pdf

[7] Kuiken T, Feuser A, Barlow A. Targeted muscle reinnervation. Taylor & Francis; 2013 [cited 2015 Jan 7]. Available from: http://www.crcnetbase.com/doi/book/10.1201/b15079

[8] Davis RW (1993). Phantom sensation, phantom pain, and stump pain. Arch Phys Med Rehabil..

[9] Sherman RA, Sherman CJ, Gall NG. A survey of current phantom limb pain treatment in the United States. Pain [Internet]. 1980;8:85–99. Available from: http://content.wkhealth.com/linkback/openurl?sid=WKPTLP:landingpage&an=00006396-198002000-00008 10.1016/0304-3959(80)90092-5 6988765

[10] Dellon AL, Aszmann OC (2020). In musculus, veritas? Nerve “in muscle” versus targeted muscle reinnervation versus regenerative peripheral nerve interface: historical review. Microsurgery..

[11] Hart SE, Kung TA (2020). Novel approaches to reduce symptomatic neuroma pain after limb amputation. Curr Phys Med Rehabil Reports Springer.

[12] Dumanian GA, Potter BK, Mioton LM, Ko JH, Cheesborough JE, Souza JM (2019). Targeted muscle reinnervation treats neuroma and phantom pain in major limb amputees: a randomized clinical trial. Ann Surg.

[13] Woo SL, Kung TA, Brown DL, Leonard JA, Kelly BM, Cederna PS (2016). Regenerative peripheral nerve interfaces for the treatment of postamputation neuroma pain. Plast Reconstr Surg - Glob Open..

[14] Kubiak CA, Kemp SWP, Cederna PS (2018). Regenerative peripheral nerve interface for management of postamputation neuroma. JAMA Surg Am Med Assoc.

[15] Kuiken TA, Childress DS, Zev RW (1995). The hyper-reinnervation of rat skeletal muscle. Brain Res Elsevier.

[16] Kuiken T, Dumanian G, Lipschutz R, Miller LA, Stubblefield K (2004). The use of targeted muscle reinnervation for improved myoelectric prosthesis control in a bilateral shoulder disarticulation amputee. Prosthet Orthot Int..

[17] Souza JM, Cheesborough JE, Ko JH, Cho MS, Kuiken TA, Dumanian GA (2014). Targeted muscle reinnervation: a novel approach to postamputation neuroma pain. Clin Orthop Relat Res..

[18] Lanier ST, Jordan SW, Ko JH, Dumanian GA (2020). Targeted muscle reinnervation as a solution for nerve pain. Plast Reconstr Surg..

[19] Santosa KB, Oliver JD, Cederna PS, Kung TA (2020). Regenerative peripheral nerve interfaces for prevention and management of neuromas.

[20] Melzack R (1987). The Short-form McGill Pain Questionnaire. Pain.

[21] Ortiz-Catalan M, Guðmundsdóttir RA, Kristoffersen MB, Zepeda-Echavarria A, Caine-Winterberger K, Kulbacka-Ortiz K (2016). Phantom motor execution facilitated by machine learning and augmented reality as treatment for phantom limb pain: a single group, clinical trial in patients with chronic intractable phantom limb pain. Lancet..

[22] Pollard CA (1984). Preliminary validity study of the Pain Disability Index. Percept Mot Skills..

[23] Herdman M, Gudex C, Lloyd A, Janssen M, Kind P, Parkin D (2011). Development and preliminary testing of the new five-level version of EQ-5D (EQ-5D-5L). Qual Life Res.

[24] Nicholas MK (2007). The pain self-efficacy questionnaire: taking pain into account. Eur J Pain Eur J Pain.

[25] Nicholas M, McGuire B, Asghari A (2015). A 2-item short form of the pain self-efficacy questionnaire: development and psychometric evaluation of PSEQ-2. J Pain.

[26] Sullivan MJL, Bishop SR, Pivik J (1995). The Pain Catastrophizing Scale: development and validation. Psychol Assess.

[27] McWilliams LA, Kowal J, Wilson KG (2015). Development and evaluation of short forms of the Pain Catastrophizing Scale and the Pain Self-efficacy Questionnaire. Eur J Pain.

[28] Lendaro E, Hermansson L, Burger H, Van der Sluis CK, McGuire BE, Pilch M (2018). Phantom motor execution as a treatment for phantom limb pain: protocol of an international, double-blind, randomised controlled clinical trial. BMJ Open.

[29] Sullivan MJL, Thorn B, Haythornthwaite JA, Keefe F, Martin M, Bradley LA (2001). Theoretical perspectives on the relation between catastrophizing and pain. Clin J Pain..

[30] Jones SMW, Lange J, Turner J, Cherkin D, Ritenbaugh C, Hsu C (2016). Development and Validation of the EXPECT Questionnaire: assessing patient expectations of outcomes of complementary and alternative medicine treatments for chronic pain. J Altern Complement Med..

[31] Wright A (2016). REDCap: A Tool for the Electronic Capture of Research Data. J Electron Resour Med Libr.

