# Correction: Surgical treatments for postamputation pain: study protocol for an international, double-blind, randomised controlled trial

**DOI:** 10.1186/s13063-023-07626-0

**Published:** 2023-10-09

**Authors:** Emily Pettersen, Paolo Sassu, Carina Reinholdt, Peter Dahm, Ola Rolfson, Anders Björkman, Marco Innocenti, Francesca Alice Pedrini, Juan Manuel Breyer, Aidan Roche, Andrew Hart, Lorraine Harrington, Adil Ladak, Hollie Power, Jacqueline Hebert, Max Ortiz-Catalan

**Affiliations:** 1 Center for Bionics and Pain Research, Mölndal, Sweden; 2 Department of Electrical Engineering, Chalmers University of Technology, Gothenburg, Sweden; 3 Center for Advanced Reconstruction of Extremities, Sahlgrenska University Hospital, Mölndal, Sweden; 4 Department of Orthoplastic, IRCCS Istituto Ortopedico Rizzoli, Bologna, Italy; 5 Department of Hand Surgery, Institute of Clinical Sciences, Sahlgrenska Academy, University of Gothenburg, Sahlgrenska University Hospital, Gothenburg, Sweden; 6 Department of Anaes- Thesia and Intensive Care, Institute of Clinical Sciences, Sahlgrenska Academy, University of Gothenburg, Sahlgrenska University Hospital, Gothenburg, Sweden; 7 Department of Orthopaedics, Institute of Clinical Sciences, Sahlgren- Ska Academy, University of Gothenburg, Gothenburg, Sweden; 8 Department of Orthoplastic, IRCCS Istituto Ortopedico Rizzoli, University of Bologna, Bolo-Gna, Italy; 9 Department of Orthopaedic, Hand Unit, Worker Hospital, Santiago, Chile; 10 College of Medicine and Veterinary Medicine, The Queen’s Medical Research Institute, The University of Edinburgh, Edinburgh, UK; 11 Canniesburn Plastic Surgery Unit, Glasgow Royal Infrmary, 84 Castle Street, Glasgow, G40SF UK; 12 College of Medicine, Veterinary & Life Sciences, The University of Glas- Gow, University Avenue, Glasgow, G12 8QQ UK; 13 Department of Anaesthesia, St John’s Hospital at Howden, NHS Lothian, Livingston, UK; 14 Division of Plastic Surgery, Department of Surgery, Faculty of Medicine and Dentistry, University of Alberta, Edmonton, AB Canada; 15 Department of Medicine, University of Alberta, Edmonton, AB Canada; 16 Bionics Institute, Melbourne, Australia

**Correction: BMC Trials 24, 304 (2023)**

**https://doi.org/10.1186/s13063-023-07286-0**

Following publication of the original article [1], we have been informed that one exclusion criteria needs to be changed:

- “Prior surgeries to address postamputation pain” should read “Prior RPNI or TMR surgery of the nerve to be treated (with painful neuroma) to address postamputation pain.”
- In patient selection (last sentence in paragraph “Distinguishing residual limb pain, neuroma pain, and phantom limb pain” on page 6), “The following methods should be applied to document the presence of neuroma pain: compatible symptomatology, Tinel's sign test, imaging (ultrasound or MRI), and nerve block.” Should read “At least one of the following methods should be applied to document the presence of neuroma pain: compatible symptomatology, Tinel's sign test, imaging (ultrasound or MRI), or nerve block.”

The sponsor has been changed in the trial from Chalmers University of Technology to Center for Bionics and Pain Research. Therefore, the disclaimer was also updated.

The original article has been corrected.

## References

[1] Pettersen E (2023). Surgical treatments for postamputation pain: study protocol for an international, double-blind, randomised controlled trial. BMC Trials.

