# Supplementary material for: Surgical treatments for postamputation pain: study protocol for an international, double-blind, randomised controlled trial
Source: Trials. 2023 May 2;24:304. doi: 10.1186/s13063-023-07286-0 (PMC10155377; doi:10.1186/s13063-023-07286-0)
Supplement: Supplementary file 1 — Additional file 1. Pre-screening questionnaire, pdf. The questionnaire contains general questions regarding amputation situation of the potential research participant including questions about amputation, prosthetic situation, pain, and general health. [file 13063_2023_7286_MOESM1_ESM.pdf]

Pre-screening form

This is a form for you who are interested in participating in our research on the surgical treatment of pain after amputation. In this form, we ask you to answer relevant questions regarding your situation. We expect it to take approximately 5 minutes to complete.

By submitting the form, you agree that your data will be stored and used exclusively by relevant staff in the research group. The information will only be used for the purpose of determining eligibility to participate in the research conducted by the group.

Contact information

Name ______________________

Mobile phone ______________________

Email ______________________

City ______________________

Amputation

Date of amputation ______________________

Amputation cause
Ex. trauma, cancer, diabetes, or other reasons ______________________

Amputation level
Ex. transfemoral amputation ______________________

Residual limb length
Ex. 10 cm ____________ cm

Prosthetic use

Do you use a prosthesis? ☐ Yes ☐ No

If Yes, continue with next question, if No go to the last question on the page.

Type of prosthesis ☐ Passive ☐ Myoelectric ☐ Other

If other, what type: ______________________

How often do you use your prosthesis?

☐ More than 12 hours per day

☐ Couple of hours per day

☐ One time during the day

☐ Couple of hours per week

☐ Couple of times per month

☐ Never

How satisfied are you with your prosthesis?

☐	☐	☐	☐	☐	☐	☐	☐	☐	☐	☐
0	1	2	3	4	5	6	7	8	9	10

Not at all satisfied — Fully satisfied

When was your last prosthetic fitting? ______________________

Why don't you use a prosthesis? ______________________

Pain

What type of pain do you experience?

☐ Residual limb pain ☐ Phantom limb pain

What is the maximum pain intensity you have experienced?

☐	☐	☐	☐	☐	☐	☐	☐	☐	☐	☐
0	1	2	3	4	5	6	7	8	9	10

No pain — Unbearable pain

What is the average pain intensity you have experienced in the last two weeks?

☐	☐	☐	☐	☐	☐	☐	☐	☐	☐	☐
0	1	2	3	4	5	6	7	8	9	10

No pain — Unbearable pain

How often do you experience pain?

☐ Constantly

☐ Couple of hours per day

☐ Couple of times during the day

☐ Couple of times during the week

☐ Couple of times during the month

☐ Never

Which pain treatments have you previously received?

Fill in all the treatments you have previously tried.

☐ Talk therapy ☐ Painkillers

☐ AR or VR ☐ Physical activity

☐ TENS ☐ Other treatments

If others, which: ______________________________

General health

Do you smoke? ☐ Yes ☐ No

Do you have high blood pressure? ☐ Yes ☐ No

Do you have diabetes? ☐ Yes ☐ No

Other questions

Do you participate in another clinical study? ☐ Yes ☐ No

If yes, which type of study: ______________________________

Is there something else you want us to know?

