# Supplementary material for: Surgical treatments for postamputation pain: study protocol for an international, double-blind, randomised controlled trial
Source: Trials. 2023 May 2;24:304. doi: 10.1186/s13063-023-07286-0 (PMC10155377; doi:10.1186/s13063-023-07286-0)
Supplement: Supplementary file 2 — Additional file 2. Information and consent form, pdf. Written information about the study that potential research participants interested in participation will receive and consent form that the research participant and clinical investigator sign. [file 13063_2023_7286_MOESM2_ESM.pdf]

Information to research participants

We would like to ask you if you would like to participate in a research project named "Surgical treatments for postamputation pain in amputees". This document contains information about the project and what it means to participate. The study is performed by Chalmers University of Technology in collaboration with Region Västra Götaland and the University of Gothenburg. The study is reviewed by the Swedish Ethical Review Authority.

What is the project and why would you like me to participate?

Major amputation is a life-changing event for the patient. Beyond the loss of motor function, the majority of individuals with amputations experience some type of postamputation pain which can continue for weeks, months, years or throughout the individual's life. The most commonly reported pains can be classified as residual limb pain, neuroma pain, and phantom limb pain, all of which have been shown to infringe on the person's daily life and negatively influence their social activities, work ability and sleep.

The overall aim of this project is to investigate and compare three different surgical techniques used as treatments for postamputation pain. All three surgery options are included in clinical practice. By participating in this study, you will not only contribute to more knowledge in the field, but hopefully experience a reduction in your postamputation pain. You will be randomised to one of the three surgical techniques and you will not know which treatment you received up to 12 months post-surgery.

How is the study performed?

The study requires you to participate in 10 visits, including 3 visits prior to surgery, 1 visit for surgery, and thereafter 6 follow-up visits spread out over a 4-year period post-surgery. All visits will primarily take place at Sahlgrenska University Hospital in Mölndal, and some visits will be performed via phone or video call.

At each visit, you will be asked to participate in different tasks such as answering questionnaires, participating in interviews, and undergoing physical examinations. In one of the visits, the surgical procedure will take place. Information regarding the surgery and what happens at each visit is provided below.

- **Pre-screening.** You will be asked to answer general questions regarding your health situation via a pre-screening questionnaire either in paper or digital format, including questions regarding your type and time of amputation, previous treatments, medications, and comorbidities.
  Time: 5-10 min

- **Screening visit.** You will be asked to attend an in-person visit where a physician will perform a physical examination of your residual limb, and you will have the chance to ask questions. You will also be asked to answer relevant questionnaires. The physical examination includes a structured pain localisation at the residual limb by applying a digital pressure at different locations.
  Time: 1-2 h

- **Baseline period.** Consists of three phone/video calls and during a period of 1-2 weeks. You will be called at three random times and asked to indicate your current residual limp pain, neuroma pain, and phantom limb pain on a scale of 0-10.
  Time: 5 min

- **Surgery.** You will undergo the study-specific surgery to which you are assigned. The surgery will take place at Sahlgrenska University Hospital in Mölndal. The surgery will take 1-3 h and you can leave the hospital the same day, unless the physician decides that you should stay overnight. Four weeks prior to surgery you are not allowed to drink alcohol, and you must fast starting at latest midnight the day before. One of the surgical treatments requires a muscle donor from the inner part of your thigh, resulting in an extra scar in addition to the scar at your residual limb.
  Time: 1-3 h (surgery), 1 day (hospital stay), the physician can decide that you need to stay overnight

- **Follow-up (1-, 3-, 6- and 12-months post-surgery, as well as 2- and 4-years post-surgery).** We will ask you to answer questionnaires and a physical examination by the physician. The physical examination will also include general control of wound-healing (besides pain localisation). At the 12-months follow-up visit you will be asked to participate in a semi-structured interview with opened-ended questions. In the interview, you determine how much information you would like to share, and it will be recorded for analysis purposes.
  Time: 2-3 h

Potential risks of participating in the study

The intervention in this study is surgery, which you as a participant would normally be provided as an option for pain management as part of your standard healthcare. Thus, the risks due to the intervention consist of general surgical consequences such as those due to anaesthesia, the possibility for superficial or deep bacterial infections, and delayed wound healing. There are no known additional risks to any of the surgical treatments investigated in this study, however, one of the surgical techniques requires a second incision as part of the surgical procedure, which provides an additional opportunity for infection or delayed wound healing. Even though the surgical

approaches in this study have shown good potential for post-amputation pain reduction in the literature, there is also a minor risk that the treatment results in no change or a worsened pain situation. In this situation, the clinical investigator will provide alternative pain management options, a consequence of which can be increased consumption of painkillers.

Risks associated with study-specific assessments:

- **Physical examination.** There are no direct risks associated with the physical examination. However, the localisation of the pain is performed by application of a pressure at the residual limb which can be perceived as unpleasant.

- **Questionnaires and semi-structured interviews.** There are no known risks associated with completing the questionnaires and participating in semi-structured interviews. Data will be handled to ensure participant integrity.

Are there any benefits to participating in this study?

If the treatment is successful, you may experience complete or partial reduction in your residual limb pain. If you also experience neuroma pain or phantom limb pain, there is a potential that the pain intensity is reduced as well. This benefit can potentially result in decreased infringement of daily activities such as social events, occupation, and sleep, thus increased quality of life. Furthermore, other potential benefits from reduced pain level are the possibility for a usage decrease of pain medication and a usage increase of a prosthesis.

Moreover, the results of the study may lead to better knowledge in the field and can potentially help other persons who suffer post-amputation pain.

What happens to my personal data?

During the study, we will collect data about your personal contact details, birthday, sex, medical history record, general quality of life, as well as results of the investigations in the study including questionnaire answers, sound recordings from interviews, and data from the physical examinations. The purpose of collecting this data is research. As research is considered to be in the public interest, it is the legal basis for the handling of personal data. Your information is confidential, and no unauthorized person has access to the data. Collected study specific data will be stored securely at our facilities and processed by relevant research personnel who are committed to protecting the confidentiality of your personal information. Your data will be stored under a pseudonym in password-protected files. Only relevant research personnel have access to the password-protected list where the pseudonyms are matched with your name.

A clarification regarding your data: research data is the data collected during study specific assessments and this data will be saved and stored unidentifiable at local servers, as described above. In addition, clinical data will be recorded according to clinical routine and this data will be linked to your name and social security number. This clinical data will only be accessible for relevant hospital personnel and it will not be included in the research study.

According to the EU's data protection regulations, you have the right to ask without cost for information regarding which data about you will be handled in the study, and if needed, to correct any potential errors. You can also request that the personal data about you is deleted, and that the treatment of your personal data is restricted. If you would like to get more information about your personal data, you can contact the researchers responsible for the study (contact information below). The Data protection officer at Chalmers University of Technology, Gothenburg, can be reached at dataskydd@chalmers.se. If you are dissatisfied with how your personal data is handled, you have the right to file a complaint with the Swedish Authority for Privacy Protection, which is the regulating authority.

Your personal data will only be used for the purposes provided above. They can only be processed for other purposes if you leave a new consent and/or a new approval is obtained from the Swedish Ethical Review Authority.

In addition to research staff, a person appointed by the clinic or an official may compare collected study data with your medical record for quality purposes to see if the study has been carried out correctly. These people must sign a confidentiality agreement before they can access your medical record. By signing the consent, you give your permission for this insight into your patient record.

Study data is saved at least 10 years after the study is completed, or as long as is required by law.

How do I get information about the result of this study?

The results will be published in scientific journals and presented at national or international scientific meetings. Nothing that you share with us will be shared with anyone outside of the research group, and nothing will be attributed to you by name. Some of the results may be shared with another research team but this would be de-identified data which means that they cannot recognize you from the data. Whenever possible, statistics at a group level will be presented and no specific individual will be able to be identified.

Insurance and compensation

You will be insured by "Patientskadeförsäkringen" through the Patient Injury Act, for the surgical intervention and the physical examinations. For all other study-specific activities, you will be insured by Chalmers Liability Insurance.

You will not be compensated for participating in the study. Healthcare will cover potential costs related to the surgery, including travel, and the research team will cover potential costs related to the research-specific assessments, including travel.

Participation is voluntary

Your participation is voluntary, and you can choose to cancel your participation at any time. If you choose not to participate or want to cancel your participation, you do not have to state why, nor will it affect your future care or treatment.

If you wish to cancel your participation, please contact one of the researchers responsible for the study (contact information below).

Responsible for the study

The researchers responsible for the study are Dr. Carina Reinholdt (clinical investigator) and Professor Max Ortiz Catalan (principal investigator).

Carina Reinholdt, MD, PhD
Telephone: +46(0)700- 85 26 12
E-mail: carina.reinholdt@vgregion.se

Max Ortiz Catalan, PhD
Telephone: +46(0)708- 46 10 65
E-mail: maxo@chalmers.se

Consent to participate in the study

Statement from the participant:

I have received verbal and written information about the study and have had the chance to ask questions. My questions have been answered satisfactorily and I accept to participate in this study. I may keep the written information.

☐ I voluntarily consent to participate in this study.

☐ I agree that information about me is processed as described in the information to research participants.

Place and date	Signature

Statement from the researcher/person who takes consent:

I have carefully read the above information for the potential participant and to the best of my ability made sure that the participant understands the purpose and procedures of the study.

I confirm that the participant was given an opportunity to ask questions about the study and that all questions asked by the participant have been answered correctly and to the best of my ability.

I confirm that the individual has not been forced to give consent and the consent has been given freely and voluntarily.

A copy of this form has been given to the participant.

Place and date	Signature
