# Supplementary material for: Surgical treatments for postamputation pain: study protocol for an international, double-blind, randomised controlled trial
Source: Trials. 2023 May 2;24:304. doi: 10.1186/s13063-023-07286-0 (PMC10155377; doi:10.1186/s13063-023-07286-0)
Supplement: Supplementary file 3 — Additional file 3. Mirror therapy protocol, pdf. Instructions on how to perform mirror therapy at home by Alberta University Hospital. [file 13063_2023_7286_MOESM3_ESM.pdf]

Mirror Therapy Protocol

Equipment

- Mirror - any mirror big enough to see the full healthy limb (i.e., wall mirror, mirror box or larger desk mirror)
- Comfortable chair or wheelchair
- Quiet space with minimal to no distractions

Selection criteria

- Limb amputation on one side only, leg or arm, at any level.
- The limb on the opposite side must be healthy, without a wound or a scar that could modify the perception of the other limb.
- Have the cognitive ability to understand the mirror therapy principle and application.
- Supervision by a trained professional is recommended if the person:
    - Has anxiety or physical image problems;
    - Has a history of previous complex regional pain syndrome;
    - Has a history of "psychosis" (a disorder of thought or the mind involving loss of contact with reality, diagnosed by a psychological health professional)
    - Has a diagnosed brain injury

Basic Tips

- Limit the length of the treatment to a 15 min maximum. Stop the treatment if you lose focus and come back to it when able
- Ensure that the position of the mirror results in realistic look of the reflected arm
- Do not have anything else visible in the reflection (i.e., door or items on the table)
- If the phantom limb feels like it is hot, try holding an ice pack with the intact limb so that it looks like holding two ice packs
- If the phantom limb feels itchy, try rubbing the area that is itchy on the phantom limb
- It may take several sessions before you gain some control of the phantom limb
- Build a routine and try and do the mirror therapy in the same place and around the same time each day

Before starting treatment

1. Remove jewelry and/or a watch from the intact arm
2. Cover any forearm tattoos (if present) with a long sleeved shirt
3. Intact hand is placed in front of the mirror and the arm/leg with the amputation is not visible as it is placed behind the mirror or where the mirror is mounted (i.e. behind a door)
4. Take a few deep breaths prior to starting the therapy to clear you mind

How to begin

1. Always start in the position of the phantom limb and work out from there
2. Slow controlled movements are key. The goal is to get to a relaxed position and not full movement of the limb
3. Ensure that the phantom limb has some movement in the direction that you are attempting to move the limb
4. Do not attempt to move the arm/leg behind the mirror to start, focus on the visual input
5. Move one body part at a time
6. Work on getting some control of the phantom limb. Any movement is a good start!

How to progress

7. Start incorporating simple movements such as individual finger opening/closing, touching the thumb to each finger, wrist flexion/extension, slow waving motion and elbow flexion/extension
8. Work on attempting to move the muscles of the arm with the amputation in coordination to the movements being attempted in the mirror
9. When able to control more than one joint of the phantom limb, work on moving two body parts at the same time
